# The Role of PIEZO1 in Urinary Bladder Function and Dysfunction in a Rodent Model of Cyclophosphamide-Induced Cystitis

**DOI:** 10.3389/fpain.2021.748385

**Published:** 2021-10-12

**Authors:** Katharine I. K. Beča, Beatrice M. Girard, Thomas J. Heppner, Grant W. Hennig, Gerald M. Herrera, Mark T. Nelson, Margaret A. Vizzard

**Affiliations:** ^1^Department of Neurological Sciences, The Larner College of Medicine, University of Vermont, Burlington, VT, United States; ^2^Department of Pharmacology, The Larner College of Medicine, University of Vermont, Burlington, VT, United States

**Keywords:** mechanosensation, calcium signaling, micturition, interstitial cystitis (IC)/bladder pain syndrome (BPS), PIEZO1

## Abstract

In the urinary bladder, mechanosensitive ion channels (MSCs) underlie the transduction of bladder stretch into sensory signals that are relayed to the PNS and CNS. PIEZO1 is a recently identified MSC that is Ca^2+^ permeable and is widely expressed throughout the lower urinary tract. Recent research indicates that PIEZO1 is activated by mechanical stretch or by pharmacological agonism via Yoda1. Aberrant activation of PIEZO1 has been suggested to play a role in clinical bladder pathologies like partial bladder outlet obstruction and interstitial cystitis/bladder pain syndrome (IC/BPS). In the present study, we show that intravesical instillation of Yoda1 in female Wistar rats leads to increased voiding frequency for up to 16 hours after administration compared to vehicle treatment. In a cyclophosphamide (CYP) model of cystitis, we found that the gene expression of several candidate MSCs (*Trpv1, Trpv4, Piezo1*, and *Piezo2*) were all upregulated in the urothelium and detrusor following chronic CYP-induced cystitis, but not acute CYP-induced cystitis. Functionally with this model, we show that Ca^2+^ activity is increased in urothelial cells following PIEZO1 activation via Yoda1 in acute and intermediate CYP treatment, but not in naïve (no CYP) nor chronic CYP treatment. Lastly, we show that activation of PIEZO1 may contribute to pathological bladder dysfunction through the downregulation of several tight junction genes in the urothelium including claudin-1, claudin-8, and zona occludens-1. Together, these data suggest that PIEZO1 activation plays a role in dysfunctional voiding behavior and may be a future, clinical target for the treatment of pathologies like IC/BPS.

## Introduction

Proper micturition requires the urinary bladder to act as a reservoir for storage, and a pump for the elimination of urine. This process relies on the presence of stretch activated channels (SACs), also known as mechanosensitive ion channels (MSCs), to transduce the mechanical distention of the bladder wall during accommodation of urine into sensory signals that are relayed to the central nervous system to perceive bladder fullness.

Several MSCs, like TRPV1 and TRPV4, are expressed in the urothelial cell layer of the urinary bladder wall and are crucial for the ultimate sensory detection of bladder distention (1, 2). Upon stretch-induced activation, these channels open a non-selective cationic pore to allow the influx of ions, including calcium (Ca^2+^), into the urothelial cell (3–5). This influx of Ca^2+^ leads to a release of several neuromodulators, including ATP, which then bind to receptors on afferent nerve terminals in the deeper layers of the bladder wall. Evidence of this comes from studies that show the addition of TRPV1 and TRPV4 agonists onto urothelial cells increases ATP release from these cells, while the addition of antagonists decreases ATP release (1, 5–7). This decrease in ATP release following antagonist application is mirrored by impaired stretch evoked responses in bladders from *Trpv1*^−/−^ and *Trpv4*^−/−^ mice along with low-amplitude bladder activity resulting in increased bladder capacity (1, 2). Though this mechanism of mechanotransduction is well-established in TRP channels, over the last decade, a novel MSC was identified in the urinary bladder called PIEZO1.

PIEZO1 belongs to a family of Ca^2+^ permeable ion channels that also includes PIEZO2. Both PIEZO1 and PIEZO2 are large, mechanically gated ion channels that were discovered in the Neuro2A mouse cell line in 2010. Since their discovery, numerous research studies have sought to understand the contribution of these channels to physiological stimuli like touch, pressure, and stretch (8). Though PIEZO1 and PIEZO2 share ~50% identity, they are functionally distinct and are differentially expressed in tissues throughout the body. For example, *Piezo1* gene expression is found in high levels in the skin, kidneys, lungs, and urinary bladder, while *Piezo2* is predominantly expressed in sensory neurons in the trigeminal ganglia, dorsal root ganglia (DRG), and Merkel cells, as well as in lower levels in the lungs and urinary bladder (8).

The present studies primarily focus on PIEZO1 due to its expression profile in the urinary bladder as well as its ability to be pharmacologically manipulated by a commercially available agonist (Yoda1) (9–12). Previous studies have already shown *Piezo1* gene expression throughout all three layers of the bladder wall (urothelium, lamina propria, and detrusor) (13). Other studies have found similar results, however, by co-staining with cytokeratin-7, some have observed that *Piezo1* gene and PIEZO1 protein expression is predominant in cells of the urothelium (14). Like other MSCs in the urothelium, upon stretch, activation of PIEZO1 leads to an increase in intracellular calcium concentration ([Ca^2+^]_*i*_) which results in ATP release. In support of this, in cultured urothelial cells, mechanical stretch stimulation leads to an increase in [Ca^2+^]_*i*_ and release of ATP that was significantly decreased in cells treated with siRNA against *Piezo1* or the non-selective MSC inhibitor, GsMTx4 (14).

In addition to their role in bladder function, PIEZO1 and other MSCs have been shown to contribute to bladder dysfunction in models of interstitial cystitis/bladder pain syndrome (IC/BPS) (7, 15–18). IC/BPS affects as many as 3.5-8.6 million women and 2.4-5.3 million men in the United States (19, 20) and is characterized by the hallmark symptoms of increased urinary urgency and frequency accompanied by pelvic pain, which typically presents as increased hyperalgesia and/or allodynia (21, 22). Currently, the etiology is unknown and there are few options for treatment for patients with this pathology.

In this paper, we show a functional role for PIEZO1 activation on micturition in female rats using an *in vivo* intravesical instillation of Yoda1 (0.4 mg/kg). Further, we show that MSC mRNA expression, including *Piezo1*, increases in the urothelium and detrusor bladder wall layers with differing durations of CYP-induced cystitis. Additionally, short exposures to CYP increased urothelial Ca^2+^ activity following activation of PIEZO1. Lastly, we hypothesize that increased activation of urothelial PIEZO1 and subsequent downstream signaling may contribute to the bladder overactivity symptoms of CYP-induced cystitis specifically by altering expression of tight-junctions which may contribute to increased permeability of the urothelium.

## Methods

### Experimental Animals

The following experiments were performed in accordance with institutional and national guidelines and regulations. The University of Vermont Institutional Animal Care and Use Committee approved all experimental protocols (no. X9-020) involving animal use. Animal care was under the supervision of the University of Vermont's Office of Animal Care and Management in accordance with the Association for the Assessment and Accreditation of Laboratory Animal Care and National Institutes of Health guidelines. All efforts were made to minimize the potential for animal pain, stress, or distress.

Female Wistar rats (*n* = 5-9 per treatment group, unless otherwise noted) and female C57/BL6 mice (*n* = 5-6 per treatment group) were bred locally at the Larner College of Medicine at the University of Vermont or purchased from Charles River Laboratories. Females were used as subjects due to experiments involving drug administration via external urethral catheterization. Rats and mice were maintained under standard laboratory conditions: they were housed two per cage and maintained on a 12:12-h light-dark cycle, with free access to food and water. Estrous cycle status was not determined in these studies.

### Yoda1 Infusion Into the Urinary Bladder Using External Catheterization

Rats were anesthetized with isoflurane (2-3%) and externally catheterized using a transurethral catheter. This was performed using a lubricated 22-gauge gavage needle and gently inserting it through the urethra and into the bladder, such that it did not have contact with the bladder wall. Yoda1 (0.4 mg/kg) was infused into the bladder and remained there for 30 min (10, 12, 17, 23). A vehicle solution of 5% DMSO in 95% saline was also infused in a separate cohort of rats. After the 30-min infusion, the bladder was manually voided. The rats recovered from anesthesia for 30 min before being put into the Urovoid system to study functional bladder outcomes.

### Natural Voiding Assay Using the Urovoid System

Metabolic cages and the Urovoid system (Med Associates, St. Albans Vermont) were used to study functional voiding parameters in rats. On the day of functional testing, rats (*n* = 6-7 per group) were placed into individual metabolic cages, situated above a flask on a balance, connected to a computer with Urovoid software. Rats remained in these cages with *ad libitum* access to food and water for 24 h (h). Over that time, their urine was caught in a flask on a balance, and the computer recorded the time and weight of each void to obtain functional voiding parameters such as number of voids, intermicturition interval (the time between each void to determine voiding frequency) and the mass of each void. Volume of water consumed by each rat in 24 h was also recorded and analyzed.

Rats received two voiding assessments using the Urovoid system. First, a baseline assessment was done 7-10 days before Yoda1/vehicle infusion to assess each rat's normal voiding function. Before the start of this baseline Urovoid assessment, the rats were anesthetized with isoflurane (2-3%), externally catheterized and infused with saline for 30 min. For the second assessment, 7-10 days later at the same time of day, the same rats were intravesically infused for 30 min with Yoda1 (0.4 mg/kg) or vehicle while the rats were anesthetized (2-3% isoflurane). The voiding assessment began 30 min after the infusion. By using this method of repeated assessments, individual differences and variability were minimized since rats are compared to their own baseline voiding function. Furthermore, since baseline assessments were done after administration of anesthesia and a transurethral catheter, the effects seen in the experimental groups are not due to these variables. Rats were excluded if their volume of water intake in the second (i.e., experimental) assessment was increased by over 20% compared to baseline consumption to ensure that changes in voiding frequency from baseline were not due to changes in water consumption.

### CYP Administration and Tissue Harvest

Rats/mice were lightly anesthetized with isoflurane (2–3%) and given a single injection of CYP (150 mg/kg, i.p. for rats; 200 mg/kg, i.p. for mice) to induce acute and intermediate cystitis, or three injections (75 mg/kg, i.p. for rats and mice) every 72 h to induce chronic cystitis. For acute experiments, a cohort of rats/mice were euthanized (5% isoflurane and thoracotomy) 4 h after the CYP injection. For the intermediate experiments, rats/mice were euthanized 24 h after the CYP injection. Lastly, for the chronic CYP-treated cohort, rats/mice were euthanized 24 h after the last CYP injection on day (d) 8. The urinary bladder was immediately dissected after euthanasia.

### Tissue Dissection for mRNA Analysis

Following the tissue harvest, the urinary bladder was quickly dissected under RNase-free conditions. The bladder was cut open along the midline and pinned to a Sylgard-coated dish, and the urothelium, including the suburothelium, was removed from the detrusor muscle with fine forceps and iris scissors. The urothelium and detrusor were evaluated separately.

### RT-qPCR for Quantitative Analysis of Gene Expression

We first extracted total RNA from samples (*n* = 5-9) using Stat-60 total RNA/mRNA isolation reagent (Tel-Test B Labs, Friendswood, TX). RNA samples (2 μg) were then used to synthesize cDNA using a mixture of random hexamers and oligo(dT) primers with M-MLV reverse transcriptase (Promega). Primers (Table 1) were designed using Oligo Primer Analysis Software 7.0. We prepared the quantitative PCR standard for each transcript using the amplified cDNA products ligated directly into the pCR2.1 TOPO vector using the TOPO TA cloning kit (Invitrogen). To verify the nucleotide sequences of the inserts, we used automated fluorescent dideoxy dye terminator sequencing (Vermont Integrative Genomics Resource). All cDNA templates were diluted 10-fold (to limit the inhibitory effects of the reaction components) and assayed using Luna universal quantitative PCR master mix (New England Biolabs). We performed qPCR (7500 Fast real-time PCR system, Applied Biosystems, Foster City, CA) under standard conditions as outlined: (24) serial heating at 94°C for 2 min and (19) amplification over 45 cycles at 94°C for 15 s and 60°C for 30 s. The amplified product was exposed to SYBR green I melting analysis by ramping the temperature of the reaction samples from 60° to 95°C. We ensured the creation of a single DNA melting profile under the outlined dissociation assay conditions to confirm the amplification of a unique, single product that was free of primer dimers and other anomalous products.

**Table 1:** Sequences of forward and reverse primers for 9 genes of interest and 2 housekeeping genes used for RT-qPCR experiments.

**Gene**	**Primer sequence**
*Trpv1*	Forward: TACTTTTCTTTGTACAGTCACT Reverse: TCAATCATGACAGCATAGAT
*Trpv4*	Forward: GAGGAGAGGTCGTAGAGAGAAGAAT Reverse: ACTGGCAAGATCGGGGTCTT
*Piezo1*	Forward: GACGCCTCACAAGGAAAGC Reverse: GGGCAGCATCTATGTCATCC
*Piezo2*	Forward: GATGGCATGGAGCATCACCTAC Reverse: CCAGCAGCAGATTCGCATAGAC
*Cldn1*	Forward: GTCAATGCCAGGTATGAAT Reverse: CTAGAAGGTGTTGGCTTGG
*Cldn2*	Forward: GCATTCTCCGGGACTTCTACTC Reverse: GCAGGAAAAGCAGAGGATGAC
*Cldn4*	Forward: CAGAGAGAGATACTTTTCG Reverse: CGTTGTGTGCCGTCCAG
*Cldn8*	Forward: GGCAACCTACGCTCTTCAAA Reverse: CAGGGAGTCGTAGACCTTGC
*ZO-1*	Forward: CTGAAGAGGATGAAGAGTATTACC Reverse: TGAGAATGGACTGGCTTGG
*18S*	Forward: AGTCGCCGTGCCTACCAT Reverse: GCCTGCTGCCTTCCTTG
*L32*	Forward: CCTGGCGTTGGGATTGGTGA Reverse: GAAAAGCCATCGTAGAAAGA

*Trpv, transient receptor potential vanilloid; Cldn, Claudin; ZO-1, zona occludens-1*.

### Quantification of Mechanoreceptor and Tight Junction mRNA Expression

For data analysis, we constructed a standard curve by amplification of serially diluted plasmids containing the target sequence. The data were analyzed at the termination of each assay utilizing sequence detection software (version 1.3.1, Applied Biosystems, Norwalk, CT). In standard assays, default baseline settings were selected and the increase in SYBR green I fluorescence intensity (ΔRn) was plotted as a function of cycle number. The threshold cycle was determined by the software as the amplification cycle at which ΔRn first intersects the established baseline. Normalized data are expressed as the relative quantity of the gene of interest normalized to the relative quantity of the housekeeping gene L32 or an average of 18S and L32 housekeeping genes.

### Preparation of Intact Urothelial Sheets and Measurement of Intracellular Ca^2+^ Events

Mice were used for Ca^2+^ imaging experiments due to difficulties loading Ca^2+^ fluorescent dye into urothelial sheets procured from rats.

Harvested bladders from naïve (no CYP)/4 h/24 h/chronic (8 d) CYP-treated mice were placed in cold HEPES solution composed of (mM): 134 NaCl, 6 KCI, 10 glucose, 10 HEPES, 1 MgCl_2_, 2 CaCl_2_, 10 glucose and adjusted to pH 7.4 with NaOH. The bladder was cut open along the midline, and pinned to a Sylgard coated dish where the urothelium was separated from the suburothelium (i.e., lamina propria) and the detrusor using fine forceps and iris scissors (25). To detect Ca^2+^ events, the urothelial sheets were loaded with a Ca^2+^ sensitive fluorescent dye (10 μM Cal 520; AAT Bioquest, Inc.) for 90 min at 37°C before being placed in a physiological saline solution (PSS) composed of (mM): 119 NaCI, 4.7 KCI, 24 NaHCO_3_, 1.2 KH_2_PO_4_, 2.5 CaCl_2_, 1.2 MgSO_4_, 7 glucose and constantly bubbled with Biological Gas (95% O_2_, 5% CO_2_) to maintain pH at 7.4.

To measure Ca^2+^ activity following PIEZO1 activation, the urothelial sheets were first imaged for 90 s to establish baseline Ca^2+^ activity. The urothelium was then superfused for 10 min with Yoda1 (20 μM) followed by a 90 s recording of the same region. In cell-based assays, Yoda1 has an EC_50_ of 10-50 μM (12) and we found that a concentration of 20 μM optimized solubility of the compound and response.

Changes in Ca^2+^ activity after PIEZO1 activation were then compared to baseline Ca^2+^ activity. Vehicle controls (DMSO 0.1%) were also conducted in a separate cohort of mice (no CYP) to control for laser damage and any cellular effects of DMSO. Images were collected at a rate of 16 images/second using a Noran Oz laser scanning confocal microscope (26) and a Nikon 60x water immersion fluorescent objective (NA 1.2). Cal 520 was excited at 488 nm and the emitted fluorescence was collected at >500 nm (26). Analysis of Ca^2+^ events was performed with custom-written software (Volumetry G9: Grant Hennig).

### Analysis and Quantification of Ca^2+^ Events

Image sequences of the urothelium were captured (512 × 512 at a spatial resolution 0.689 μm per pixel and temporal resolution of 0.625 s). The files were saved as multislice TIFF files and imported into custom-written software (Volumetry G9d, GWH). Motion stabilization and background intensity normalization (debleaching) was applied as necessary followed by temporal averaging (±2 frames: total time 3.13 s) and spatial smoothing (Gauss 5 × 5 pixel, sd = 1.0) to reduce noise.

Loading of Cal520 into the urothelium is often uneven with cells displaying bright and dim baseline fluorescence intensities. This creates issues when measuring Ca^2+^ event amplitudes using the traditional F/F0 ratio, as cells with high background intensities will have smaller calculated amplitudes compared to cells with low background intensities, even though the absolute change in intensity during Ca^2+^ events may be similar.

To circumvent this limitation, we calculated Ca^2+^ event amplitude using a signal-to-noise approach where changes in intensity during Ca^2+^ events are put in relation to the variation of intensity during quiescent periods (27). The use of spatio-temporal (ST) maps calculated from active sites is used descriptively in this study to demonstrate patterns of Ca^2+^ events but was not used quantitatively due to the ovoid nature of urothelial cells that was not suited to dimensional averaging. To better measure the extent and intensity of Ca^2+^ events in ovoid cells, pixel binning (10 × 10 pixel average) was used to spatially reduce noise, then a conservative quiescence estimator (20% of the 15 m:40 s recording time = ~3 min) was used to demarcate time periods in which a cell was not active. A histogram of the intensities throughout the recording at each pixel was calculated, and then the standard deviation (SD) of the dimmest 20% of pixels was calculated (SDqmin). Quiescent periods were defined statistically as those portions of the recording that had intensities <11.0 SDqmin. Then, within the defined quiescent periods, standard deviation and average intensity (AVGq) was recalculated (SDq) enabling the movie intensities to be converted to Z-scores (Zscr: (intensity-AVGq)/SDq). A threshold was used to define active Ca^2+^ events (≥2.7 Zscrs) and remaining noise was filtered using a particle filter (≤ 3 pixels).

Contiguous pixels in Ca^2+^ events were coalesced into coordinate-based particles in each frame, then an overlap function was used between successive frames to determine the time course of Ca^2+^ events and create ST objects. All spatial, temporal, and intensity information was extracted from Ca^2+^ event ST objects including duration (s), area (μm^2^), maximum intensity (Zscr) and an integrated measure of elevated overall active Ca^2+^ presence or “output” in the cytoplasm of all cells in the field of view (FOV) we abbreviated to Zum^2^s (Zscr ∙ μm^2^ ∙ s.min^−1^). This measure multiplied number of active pixels by their size (μm^2^), their intensity (Zscr) and their duration (s). This value was normalized to per minute and is a useful way to summarize changes in Ca^2+^ activity in the entire FOV.

### Statistical Analyses

Values are presented as means ± SEM. Power and sample size were determined using G*Power statistical software. Data were compared using One-Way ANOVA, mixed model repeated measures ANOVA, and Student's paired *t*-test, as appropriate, with SAS and GraphPad Prism statistical software. For the mixed model repeated measures ANOVA, statistical test assumptions were not met, therefore, a square root transformation was used to compress variance in the analysis of the number voiding events, and a natural log transformation was used on the raw data in the analysis of intermicturition interval and void mass. Differences with *P* ≤ 0.05 are considered statistically significant.

## Results

### Activation of PIEZO1 With Yoda1 Increases Frequency of Bladder Voiding

Previous studies in unconscious rats have shown that blockade of PIEZO1 using the non-selective MSC antagonist, GsMTx4 (2.5 μM), increases the intermicturition interval (i.e., the time between voiding events) when infused directly into the bladders of naïve rats, and rats chronically treated with CYP (17). Thus, we hypothesized that activating PIEZO1 via intravesical infusion of Yoda1 directly into the bladder would recapitulate the hallmark symptom of increased voiding frequency commonly seen in patients with IC/BPS and animal models using CYP-treatment.

**Figure 1:**
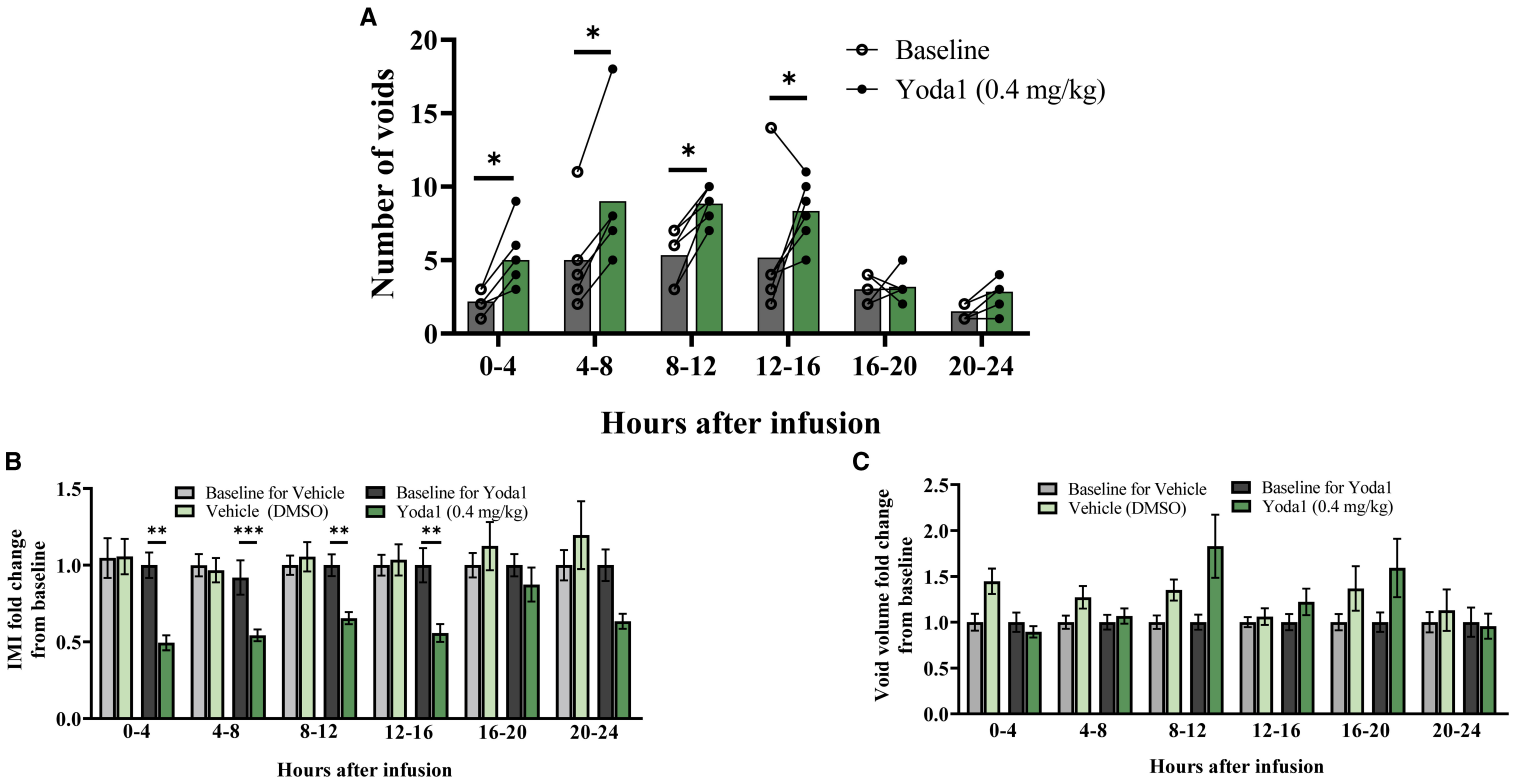
Intravesical infusion of Yoda1 alters voiding function. The voiding function of conscious, freely moving, rats was studied for 24 h post saline infusion (baseline) and compared to voiding function following Yoda1 (0.4 mg/kg) or vehicle (5% DMSO in saline) infusion. **(A)** In naïve (no CYP) female rats (*n* = 6), activation of PIEZO1 with the agonist Yoda1 significantly increases number of voids at every timepoint studied until 16 h after infusion (*P* ≤ 0.05). **(B)** This increase in number of voids is matched by a significant decrease in the IMI at the same timepoints (*P* ≤ 0.01, 0–4, 8–12, and 12–16 h, and *P* ≤ 0.001 4–8 h post infusion) compared to vehicle infusion (*n* = 7). **(C)** These changes in voiding frequency after Yoda1 infusion do not result in any changes in void mass at any timepoint studied compared to vehicle infusion. Values are means ± SEM. Statistical analyses were performed using mixed model repeated measures ANOVA on square root **(A)** or natural log **(B,C)** transformed data. Fold-change transformation in **(B,C)** was done post-statistical analysis. *P ≤ 0.05; **P ≤ 0.01; ***P ≤ 0.001.

Changes in voiding parameters after Yoda1 (*n* = 6) were compared to changes after vehicle (*n* = 7) by testing the group^*^condition interaction at each timepoint to evaluate the difference between the effects at each timepoint. Significant differences that are noted indicate a significant difference between the effects (baseline vs. vehicle compared to baseline vs. Yoda1 at each timepoint). 0-4 h after baseline infusion, rats voided an average of 3 times, which significantly increased to an average of 5 times after Yoda1 infusion (*P* ≤ 0.05; Figure 1A). From 4-8 h after Yoda1 infusion, voiding events increased to an average of 9 compared to 5 voids at baseline (*P* ≤ 0.05). From 8 to 12 and 12 to 16 h after Yoda1 infusion, rats voided an average of 9 and 8 times which were significantly more than their baseline voiding levels during each respective time period (*P* ≤ 0.05).

These changes in voiding frequency after infusion of Yoda1 corresponded with decreases in the IMI compared to vehicle such that 0-4 h after Yoda1 infusion, the IMI decreased from baseline by 50.6% (*P* ≤ 0.01). From 4 to 8 h the IMI decreased 45.7% (*P* ≤ 0.001), from 8 to 12 h the IMI decreased 34.5% (*P* ≤ 0.01), and from 12 to 16 h, the IMI decreased 44.2% compared to baseline (*P* ≤ 0.01; Figure 1B). There were no significant changes in the number of voiding events or IMI compared to baseline 16-20 or 20-24 h after Yoda1 infusion. Interestingly, even with the increase in voiding frequency, there was no statistical difference in void mass from baseline after Yoda1 administration compared to vehicle at any timepoint tested (Figure 1C). However, we did find a statistical difference in the total void mass after 24 h in rats treated with Yoda1 (28.9 g ± 11.4) compared to baseline void mass (19.1 g ± 11.4; *P* ≤ 0.05).

### MSC Gene Expression Is Increased in the Urothelium and Detrusor Following Chronic, but Not Acute, CYP-Treatment

**Figure 2:**
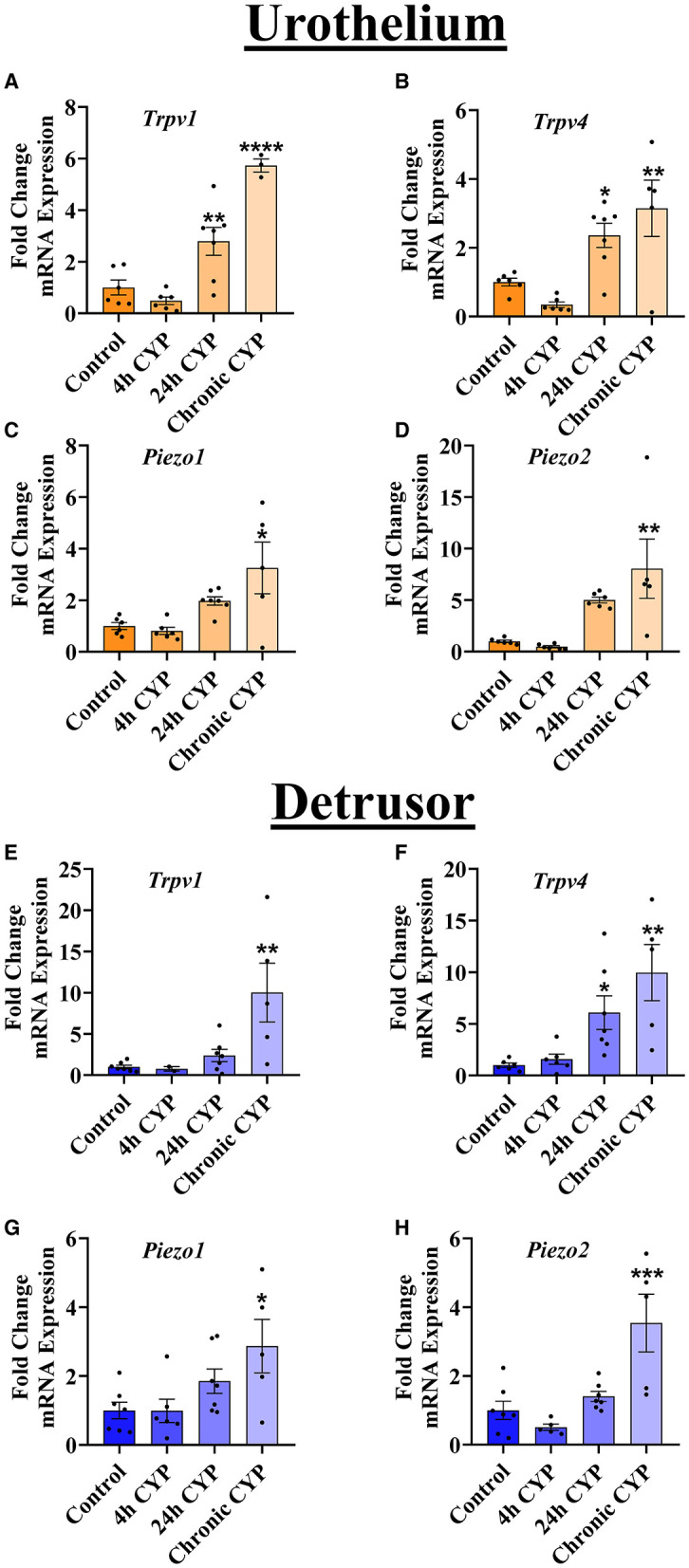
Chronic, but not acute, CYP-induced cystitis leads to increases in MSC gene expression. RT-qPCR was used to detect MSC mRNA separately in the urothelium and the detrusor layers of the urinary bladder at several timepoints of CYP treatment. **(A–D)** In the urothelium, *Trpv1* (*n* = 3–7) and *Trpv4* (*n* = 5–7) mRNA were significantly upregulated after 24 h CYP-treatment (*P* ≤ 0.01 and *P* ≤ 0.05, respectively) and after chronic CYP-induced cystitis (*P* ≤ 0.0001 and *P* ≤ 0.01, respectively). *Piezo1* (*n* = 5–7) and *Piezo2* (*n* = 5–6) were both significantly increased in the urothelium only after chronic CYP-treatment (*P* ≤ 0.05 and *P* ≤ 0.01, respectively). **(E–H)** In the detrusor, *Trpv1* (*n* = 2–7) was increased after chronic CYP-treatment (*P* ≤ 0.01), whereas *Trpv4* (*n* = 5–7) was increased following both 24 h CYP-treatment (*P* ≤ 0.05) and chronic CYP-induced cystitis (*P* ≤ 0.01). *Piezo1* (*n* = 5–7) and *Piezo2* (*n* = 5–7) mRNA were also both significantly upregulated in the detrusor following chronic CYP-induced cystitis (*P* ≤ 0.05, and *P* ≤ 0.001, respectively). Values are means ± SEM. Data were normalized to a housekeeping gene (L32) and statistical analyses were performed on raw data using a One-Way ANOVA before fold-change transformation. *P ≤ 0.05; **P ≤ 0.01; ***P ≤ 0.001; ****P ≤ 0.0001.

Due to the established importance of urothelial signaling in mechanotransduction and subsequent afferent nerve terminal activation, we hypothesized that there would be bladder tissue-specific regulation of MSC mRNA during varying durations of CYP-induced cystitis in the urothelium vs. the detrusor. Contrary to this, we observed similar regulation of all MSCs tested (*Trpv1, Trpv4, Piezo1, and Piezo2*) in both the urothelium (Figures 2A–D) and the detrusor (Figures 2E–H). Specifically, we found that compared to naïve (no CYP-treatment), chronic CYP-treatment led to increases in the mRNA expression of all MSCs examined: *Trpv1* (*P* ≤ 0.0001), *Trpv4* (*P* ≤ 0.01), *Piezo1* (*P* ≤ 0.05), and *Piezo2* (*P* ≤ 0.01) in the urothelium. Similarly, in the detrusor after chronic CYP-induced cystitis, mRNA expression of *Trpv1* (*P* ≤ 0.01), *Trpv4* (*P* ≤ 0.01), *Piezo1* (*P* ≤ 0.05), and *Piezo2* (*P* ≤ 0.001), were all upregulated. Additionally, after 24 h of CYP treatment, *Trpv1* and *Trpv4* gene expression was increased in the urothelium (*P* ≤ 0.01 and *P* ≤ 0.05, respectively) and *Trpv4* mRNA was upregulated in the detrusor (*P* ≤ 0.05). No changes in MSC expression were observed in acute (4 h) CYP treatment in the urothelium or the detrusor.

### Activation of PIEZO1 With Yoda1 Leads to a CYP-Dependent Increase in Ca^2+^ Activity in Urothelial Cells

**Figure 3:**
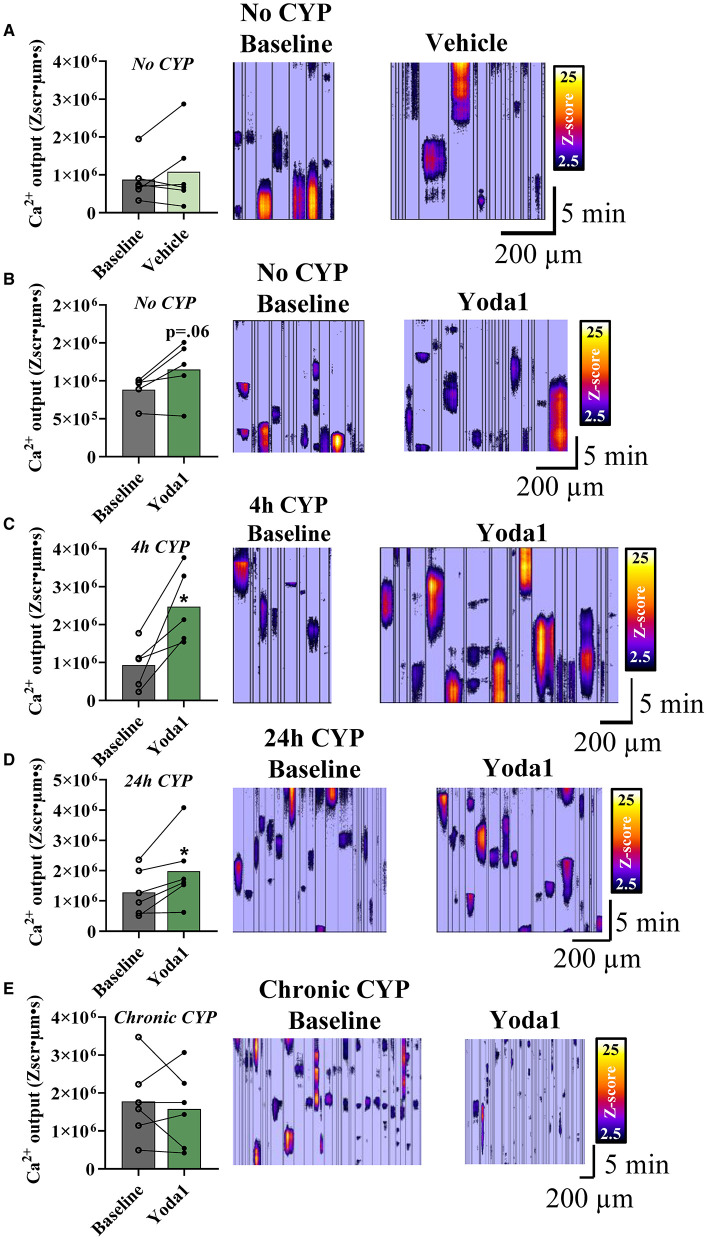
PIEZO1 activation with Yoda1 increases Ca^2+^ output in urothelial cells dependent on duration of CYP-treatment. Cells from sheets of urothelial tissue were imaged before (baseline) and after incubation with Yoda1 (20 μM) or vehicle (0.1% DMSO). **(A)** Vehicle treatment (*n* = 6) did not alter Ca^2+^ signaling in urothelial cells compared to baseline. **(B)** Activation of PIEZO1 with Yoda1 in urothelial cells also did not alter Ca^2+^ activity in naïve (no CYP) mice (*P* = 0.06; *n* = 5). **(C,D)** Urothelial cells from mice treated with 4 (*n* = 5) and 24 h (*n* = 6) of CYP-induced cystitis showed increased Ca^2+^ output when compared to their own baselines before PIEZO1 activation (*P* ≤ 0.05 for both). **(E)** Urothelial cells from chronic CYP-treatment (*n* = 6) showed variable Ca^2+^activity at baseline and no significant change in this activity following PIEZO1 activation. Values are means ± SEM. Statistical analyses were performed on using a paired Student's *t*-test. *P ≤ 0.05.

Given the changes in MSC gene expression, we utilized Ca^2+^ imaging to determine CYP-induced functional changes in PIEZO1 activity. To ensure any observed effect of Yoda1 was due to the drug itself, we first tested whether the drug vehicle (0.1% DMSO) or time affected Ca^2+^ activity. There was no change in overall Ca^2+^ activity compared to baseline after a 10-min incubation period in the vehicle solution (Figure 3A). In our experimental groups, we found that Yoda1 elicited differences in urothelial cell Ca^2+^ activity dependent on duration of CYP-treatment. In urothelial sheets from mice not treated with CYP, we found no significant difference (*P* = 0.06) in Ca^2+^ activity after Yoda1 administration (Figure 3B).

In urothelial sheets from mice that were treated with acute (4 h; Figure 3C) and intermediate (24 h; Figure 3D) durations of CYP, we found significant increases in Ca^2+^ activity compared to baseline after Yoda1 treatment. Specifically, in the 4 h CYP treatment group, the Ca^2+^ activity increased by 1.54 × 10^6^ Zum^2^s, which represents a 2.67-fold increase compared to baseline (*P* ≤ 0.05). Similarly, in the 24 h CYP treatment group, the Ca^2+^ activity increased by 7.02 × 10^5^ Zum^2^s, a 1.5-fold increase from baseline (*P* ≤ 0.05). We observed noticeable within-group variability in the Ca^2+^ activity of the chronic CYP-treated group and ultimately, there was no difference between Ca^2+^ activity at baseline and after Yoda1 in that group (Figure 3E).

Though we only demonstrated statistical significance with Yoda1 administration in two of the four experimental groups (4 and 24 h CYP treatment), we did see medium to large effect sizes, as measured by Cohen's *d* values (28), for three of the four groups compared to baseline: no CYP (*d* = 0.88), 4 h CYP (*d* = 1.86), and 24 h CYP (*d* = 0.71). Notably, these effect sizes were larger than both the vehicle control (*d* = 0.26) and the chronic CYP (*d* = 0.20) treatment groups which suggests physiological relevance of PIEZO1 activation and calcium activity in three of the four experimental groups.

### Intravesical Yoda1 Infusion Leads to Reduced Expression of Tight-Junction Genes in the Urothelium

Since there were no statistically significant changes in Ca^2+^ activity after Yoda1 in the naïve (no CYP) group, and yet we saw functional voiding changes after intravesical infusion of Yoda1 *in vivo* of naïve animals, we wanted to investigate other possible molecular changes following PIEZO1 activation with Yoda1. Previous research suggests a relationship between urothelial stretch, like with distention, and tight junction dynamics (29, 30). Therefore, we hypothesized that activation of PIEZO1 may be disrupting tight junction organization or expression in the urothelium. In this experiment we investigated gene expression changes in tight junctions that have previously been shown to be expressed in the urothelium (zona occludens-1 and claudin-2,−4, and−8) (24), as well as claudin-1 which has recently been shown to be regulated by PIEZO1 in the intestinal epithelium (30).

**Figure 4:**
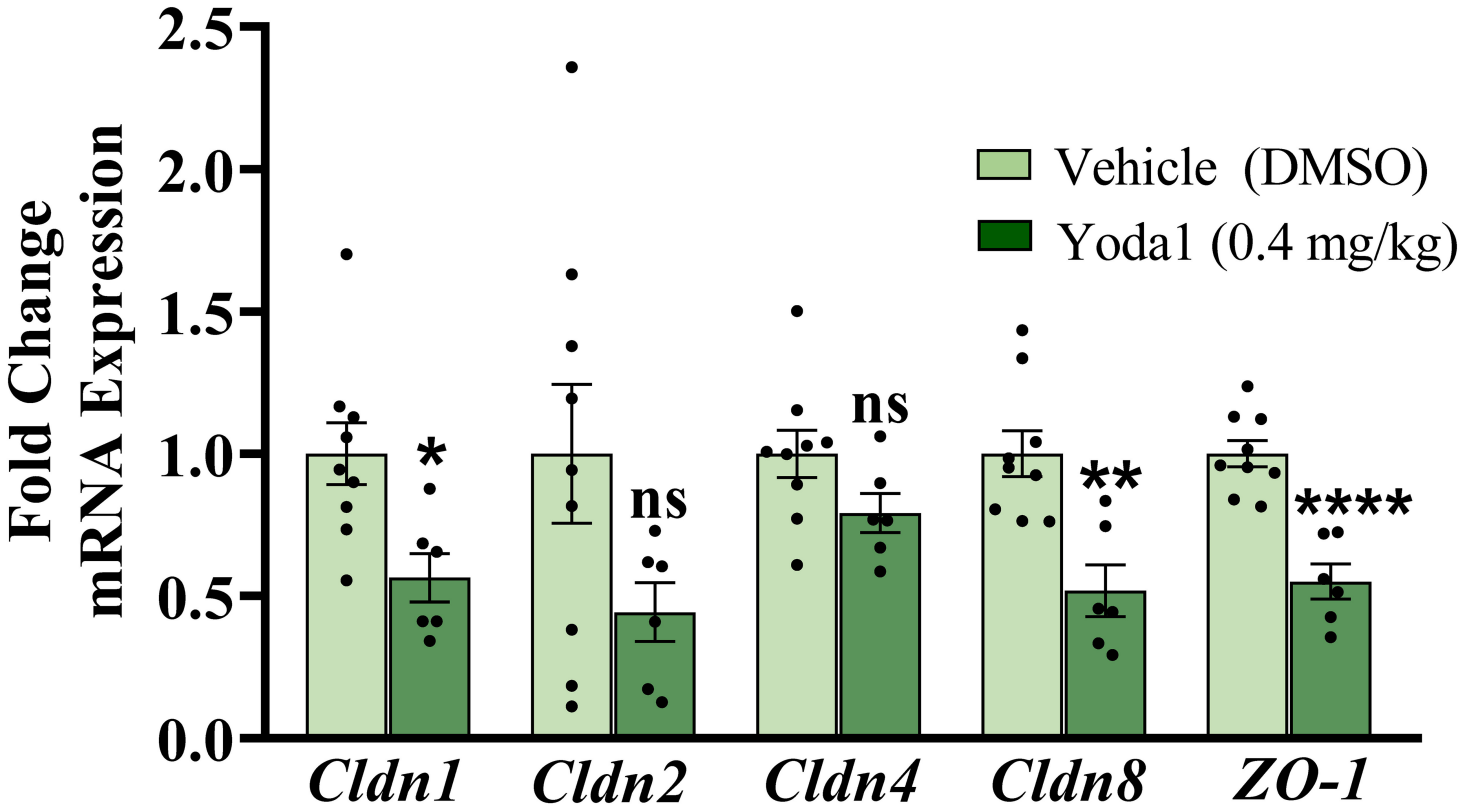
Activation of PIEZO1 results in a decrease in tight junction mRNA expression in the urothelium. Rats were externally catheterized and intravesically infused with either Yoda1 (0.4 mg/kg) or vehicle (5% DMSO in saline). RT-qPCR was used to detect expression of tight junction mRNA in the urothelium from these animals to assess possible changes with the urothelial barrier. The gene expression of several tight junctions analyzed were significantly downregulated after Yoda1 administration compared to vehicle including, *Cldn1* (*P* ≤ 0.05), *Cldn8* (*P* ≤ 0.01), and *ZO-1* (*P* ≤ 0.0001). No statistical differences were seen in *Cldn2* or *Cldn4* mRNA expression levels compared to vehicle treatment. Values are means ± SEM. Data were normalized to an average of two housekeeping genes (L32 and 18S) and statistical analyses were performed on raw data using an unpaired Student's *t*-test before fold-change transformation. *P ≤ 0.05; **P ≤ 0.01; ****P ≤ 0.0001.

Compared to rats treated with vehicle, rats treated with Yoda1 showed significant decreases in the expression of claudin-1 (*Cldn1*), *Cldn8*, and zona occludens-1 (*ZO-1*) mRNA in the urothelium (Figure 4). Specifically, *Cldn1* decreased by 43.6% (*P* ≤ 0.05), *Cldn8* decreased by 48.2% (*P* ≤ 0.01), and *ZO-1* decreased by 45.0% (*P* ≤ 0.0001). Gene expression of *Cldn2* and *Cldn4* were statistically unregulated by PIEZO1 activation when compared to vehicle.

## Discussion

The present study underscores the contribution of PIEZO1 in urinary bladder function and dysfunction. In naïve (no CYP) rats, local pharmacological activation of PIEZO1 with Yoda1 (0.4 mg/kg) induces changes in natural voiding function that persist for up to 16 h post administration. Specifically, we show that infusing Yoda1 into the bladder via non-invasive, external catheterization leads to statistically significant increases in voiding frequency and decreases in intermicturition interval (IMI) at several time points compared to baseline voiding parameters (Figure 1). We then assessed regulation of MSC gene expression in the urothelium and detrusor of rats treated with CYP-induced cystitis, an animal model of IC/BPS and a common cause of bladder dysfunction. We found that several MSC genes including *Trpv1, Trpv4, Piezo1*, and *Piezo2* were all upregulated in both layers of the bladder (urothelium and detrusor), following chronic CYP-induced cystitis, but not acute CYP-induced cystitis (Figure 2). To determine functional relevance of the changes in gene expression of *Piezo1*, we used Ca^2+^ imaging to examine differences in Ca^2+^ activity with PIEZO1 activation in rodents with different durations of CYP treatment. We found increased Ca^2+^ activity following addition of Yoda1 (20 μM) in acute (4 h) CYP treatment and intermediate (24 h) CYP treatment, but not in naïve nor chronic CYP-treatment (Figure 3). Lastly, we found that local activation of PIEZO1 led to the downregulation of several tight junction genes like *Cldn1, Cldn8*, and *ZO-1* (Figure 4). Together, these data add to our understanding of urinary bladder mechanotransduction and suggest a potential contribution of PIEZO1 activation in pathological bladder function, like IC/BPS.

To minimize the effects of inflammation on bladder function, we utilized a natural voiding assay (Urovoid), as opposed to other, more invasive, methods to functionally study PIEZO1. Notably, by infusing Yoda1 intravesically, we did not specifically activate membrane bound PIEZO1 on urothelial cells; the activation of PIEZO1 present on afferent nerve terminals, on interstitial cells in the lamina propria, and in the detrusor cannot entirely be discounted. Furthermore, due to our incubation period following Yoda1 administration, our findings indicate that downstream signaling pathways may contribute to observed changes in micturition reflex function that occur following Piezo1 activation.

Our functional studies show increases in voiding frequency in conscious, freely moving naïve (no CYP) rats, over a 24 h period after treatment with Yoda1 (Figure 1). For our *in vivo* studies, we treated rats with a dosage of Yoda1 that has been previously used in other *in vivo* studies (0.4 mg/kg) (10, 23). Our results showcase voiding changes using that dosage; however, physiological responses could also occur at different dosages. Future studies could perform a dose response curve to determine the optimal dose for Yoda1-induced changes in voiding.

Our data suggest the effects of Yoda1 treatment persist up to 16 h post-infusion of Yoda1. This attenuation may be due to the diurnal voiding cycle, since 16-24 h after Yoda1 infusion occurs during the daytime when rats are typically sleeping. Future studies could lengthen the experimental timeframe to specifically determine any diurnal effects and if changes in functional voiding continue long term due to changes in downstream signaling pathways that occur following PIEZO1 activation.

Following Yoda1 administration, we show an increase in voiding frequency compared to vehicle, though we do not see a change in voided volume during the same time intervals (Figure 1C). Because we excluded rats that drank significantly more water over the 24 h measurement period following Yoda1/vehicle administration compared to baseline, we can exclude polydipsia as an underlying factor. However, we are uncertain at this time whether the unchanged void volume is due to technical limitations of metabolic cages and measuring small voids, or a potential alteration in systemic fluid balance. We observed an increase in total void mass following the 24 h Urovoid test with Yoda1 treatment compared to baseline, which may be indicative of the latter. This may suggest that Yoda1 causes an alteration in urothelial permeability and promotes migration of interstitial fluid into the bladder. This would be corroborated with our finding that Yoda1 treatment decreases urothelial tight junction gene expression which may indicate altered urothelial permeability. Considering recent studies that show the urothelium can influence fluid volume/composition in the bladder (31–33), as well as our functional and molecular results, PIEZO1 activation may affect both fluid balance in the bladder and bladder sensation. Future functional studies could use other voiding assays (e.g., cystometry) and/or peripheral nerve recordings, to determine with certainty that PIEZO1 activation does indeed lead to changes in the afferent limb of the micturition reflex.

Lastly, though we attempted to study PIEZO1 activation in rats with CYP-induced cystitis, the within group variability due to the CYP treatment was too high to assess any changes related to PIEZO1 (data not shown). Additionally, the lack of available and specific PIEZO1 inhibitors makes studying PIEZO1 functional contributions to overactive bladder in rats challenging. Future studies will utilize siRNA or a conditional floxed *Piezo1*-knockout mice under direction of a uroplakin B promotor to knockdown *Piezo1* in the urothelium and examine the effects of local PIEZO1 inhibition on calcium activity and pathological bladder function to determine a more definitive role for PIEZO1 in CYP-induced cystitis as well as in normal bladder function.

Research shows that mRNA and protein expression of PIEZO1 increase in the whole bladder of rats after chronic CYP-induced cystitis (17). Further, a novel role for PIEZO2 as a mechanosensor has recently been elucidated in urothelial cells (34). Given this and previous data implicating TRP channels in mechanosensation in the bladder (1, 2), we hypothesized that there would be tissue-specific regulation of MSC mRNA during varying durations of CYP-induced cystitis in the urothelium vs. the detrusor. Though we did show MSC upregulation with chronic CYP treatment, we did not see specific regulation in the urothelium compared to the detrusor. Specifically, even though MSC mRNA is higher in the urothelium than the detrusor (data not shown), both layers of the bladder wall showed the same trends in MSC gene regulation with CYP-treatment duration (Figure 2).

After determining changes in gene expression, we investigated functional changes with PIEZO1 activation on a molecular level. To accomplish this, we utilized Ca^2+^ imaging in urothelial sheets within a network of urothelial cells before and after PIEZO1 activation. To conduct these studies, we made a switch in species from rat to mouse due to difficulties with isolating the rat urothelial layer from the underlying lamina propria and connective tissue, in addition to problems loading the Ca^2+^ fluorescent dye into the rat urothelial sheets. Previous research using urothelial cell cultures found that activation of PIEZO1 in response to stretch increased [Ca^2+^]_*i*_, and was attenuated upon addition of the non-selective MSC antagonist, GsMTx4 (3-5 μM), and *Piezo1* siRNA knockdown (14). Given this, we originally hypothesized that CYP-treatment would alter Ca^2+^ activity. Specifically, since we found increased expression of PIEZO1 mRNA in the urothelium with chronic CYP-treatment (Figure 2C), we hypothesized that superfusion of Yoda1 (20 μM) would increase the Ca^2+^ activity for all groups and would be most robust in the chronic CYP treated group. Our results indicated a statistically significant increase in Ca^2+^ activity only in the acute and intermediate CYP treated groups; however, we found a large effect size, as measured by Cohen's *d*, after Yoda1 administration in the no CYP experimental group as well. Notably, the chronic CYP-treatment group had variable changes in Ca^2+^ activity in response to PIEZO1 activation, which was reminiscent of the sizeable variation we saw in *Piezo1* gene expression in the chronic CYP treatment group (Figure 2). This suggests that there may be significant individual differences in the expression and function of MSCs in rodents that are treated with longer durations of CYP. Future studies are needed to observe the effect of this variability on the function and expression of PIEZO1 in the bladder. Additionally, there are several avenues for future research to examine the effects of PIEZO1 activation on Ca^2+^-induced Ca^2+^release and ATP release from the urothelium.

PIEZO1 activation-induced increases in Ca^2+^ activity and the subsequent release of ATP onto nerve terminals and may be one explanation for the contribution of PIEZO1 signaling in bladder overactivity. However, because we saw functional voiding changes after Yoda1 infusion *in vivo* but did not see corresponding statistically significant Ca^2+^ activity changes in the urothelial cells from naïve (no CYP) animals, we wanted to investigate other mechanisms that could increase voiding frequency. One such mechanism may involve downstream signaling cascades following PIEZO1 activation that lead to increases in bladder wall permeability. In the current study, we show decreases in several tight junction genes (*Cldn1, Cldn8*, and *ZO-1*) after Yoda1 administration compared to vehicle (Figure 4). These changes are similar to tight junction gene expression changes seen in the bladders of patients with IC/BPS (35). To conclude this with certainty, future research will need to assess tight junction regulation at the protein level. Furthermore, PIEZO1 signaling has been linked to the downregulation of CLDN1 via activation of the rho-associated protein kinase (ROCK) signaling pathway in the intestinal epithelium (30), therefore, specific bladder-centric studies using Ussing chambers could be conducted to further elucidate this connection and determine the cellular and permeability properties of the uroepithelial barrier following Yoda1 administration.

Lastly, though we show changes in mRNA expression and Ca^2+^ activity using a CYP model of IC/BPS, more research would need to be conducted using other models of IC/BPS to demonstrate model independence and potential clinical translatability of Piezo1 activation in voiding function. Specifically, using a psychological/physical stressor or a naturally occurring disease model, like feline interstitial cystitis, could determine if Piezo1 is indeed a possible target for therapeutic treatment (36).

In conclusion, we show both gene expression changes in the urinary bladder and functional bladder changes upon local pharmacological activation of PIEZO1 under normal and pathological conditions. The current study highlights PIEZO1 as a potential target for treatment for numerous bladder pathologies, like IC/BPS, and offers a basic mechanistic understanding of how MSCs contribute to bladder function and dysfunction.

## Data Availability Statement

The original contributions presented in the study are included in the article/supplementary material, further inquiries can be directed to the corresponding author/s.

## Ethics Statement

The animal study was reviewed and approved by the University of Vermont Institutional Animal Care and Use Committee approved all experimental protocols (No. X9-020) involving animal use. Animal care was under the supervision of the University of Vermont's Office of Animal Care and Management in accordance with the Association for the Assessment and Accreditation of Laboratory Animal Care and National Institutes of Health guidelines.

## Author Contributions

KB, BG, TH, GWH, GMH, MN, and MV conceived and designed research, interpreted results of experiments, edited and revised manuscript, and approved final version of manuscript. KB, BG, TH, and GMH performed experiments. KB, BG, TH, and GWH analyzed data. KB, GWH, and MV prepared figures. KB and MV drafted manuscript. All authors contributed to the article and approved the submitted version.

## Funding

This work was funded by National Institute of Diabetes and Digestive and Kidney Diseases Grants 5R01DK120108 and 1R01DK124580 to MV, GMH, and TH are supported by 1R01DK125543. GWH was supported by R01 NS110656-01, R35HL140027-01, R01 DK125543-01, and P20 GM135007-01 (Core C).

## Author Disclaimer

The National Institutes of Health had no role in the experiments described, including the design, data collection, and analysis of studies performed in the Vizzard laboratory, decision to publish, or preparation of the manuscript. The contents are solely the responsibility of the authors and do not necessarily represent the official views of the National Institutes of Health.

## Conflict of Interest

GMH is a scientific consultant at Med Associates, Inc, and his spouse is a co-owner of the business. The remaining authors declare that the research was conducted in the absence of any commercial or financial relationships that could be construed as a potential conflict of interest.

## Publisher's Note

All claims expressed in this article are solely those of the authors and do not necessarily represent those of their affiliated organizations, or those of the publisher, the editors and the reviewers. Any product that may be evaluated in this article, or claim that may be made by its manufacturer, is not guaranteed or endorsed by the publisher.
